# Practical Notes and Formulæ

**Published:** 1885-10-20

**Authors:** 


### PRACTICAL NOTES AND FORMULÆ.

**Ointment for Hæmorrhoids.**—For external hæmorrhoids the following ointment, suggested by Dr. Pasqua, of Florence, in the Gazetta degli Ospitali, is said to be one ot the best applications:

℞. Extract of belladonna........................grs. 5
Iodoform........................................ " 1
Acetate of lead................................. " 1
Vaseline........................................ ʒ 1

Mix. Apply three or four times a day.—*Exchange.*

A writer in Medical World says: As Dr. J. B. Gilmore, of Holly Hill, S. C., asks for experience of physicians in using hypodermic injections of carbolic acid for piles, I can say for one that I have used it for four years without a single failure or after trouble. My plan is as follows:

℞. Carbolic acid, } aa..................ʒ 1

Olive oil, }

Morphia sulphate..................................gr. 4

Mix. Inject from two to five drops with a good syringe, first annointing the pile tumor with a vaseline or other unguent.

**For Asthmatic Paroxysm.**—

℞. Tinct. lobeliæ................................℥ j

Ammon. iodidi....................................ʒ ij

Ammon. bromidi...................................ʒ iij

Syr. tolutan.....................................℥ iij

M. Sig. A teaspoonful every one, two, three or four hours.
Dr. Bartholow says that the above "gives relief in a few minutes, and sometimes the relief is permanent."—*Medical World.*

**Laxative Syrup.**—

℞. Glycerin.................... }

Syrup Rhei.................. }

Fluid extract cascara sagrada,.. } aa................℥ j.

Castor oil.................. }

Dose: Tablespoonful at bedtime.

**For Children's Dysentery.**—

℞. Hydrarg. perchloridi..........................gr. j

Pul. ipecacuanhæ.................................ʒ j

Aq. destil.......................................℥ viij

Dose from a half to a teaspoonful three to four times a day.—*Medical World.*

**Bengal Cholera Pill.**—

℞. Pulveris nigri, } aa........................gr. j

Asafœtidæ, }

Camphoræ..................................gr. ij

Mix. For one pill.—*Indiana Medical Journal.*

**Huchird's Anti-Tubercular Pills.**—Take of

℞. Creosote, }

Iodoform, }

Powdered benzoin, } aa.......................gr. ¼

Balsam of tolu, }

Mix. Make one pill. From two to four are given daily.—*Med. World.*

**Laxative Sugar.**—Under the title "laxative sugar," the Analetic suggests the following mixture:

℞. Rochelle salts..............................parts 10
Milk sugar.................................. " 30
Essential oil of lemon, sufficient quantity to flavor.

The dose is one or two heaping teaspoonfuls before breakfast.—*Medical World.*

**Application to the Gums in Dentition.**—Dr. Pierre, in the Gazette Hebdomadaire de Medicine et de Chirurgie, of 24th July, 1885, recommends the following as an anodyne in dentition:

℞. Hydrochlorate of cocain...........10 centigrammes
Simple syrup......................10 grammes
Tincture saffron..................10 drops

Mix. The gums should be gently rubbed with this syrup several times during the day.—*Medical Age.*

**Toothache Drops.**—

℞. Choral hydrate.......................20 grains
Refined camphor.......................15 grains
Chloroform............................ ½ drachm
Tinct. aconite root................... 5 drops
Oil of cloves.........................10 drops
Tinct. opium..........................20 drops

M.—*Druggists' Circular.*

**Bright's Disease.**—A good prescription for this disease should run thus:

℞. Citrate potassium...........................ʒ i

Chloroform..................................*m* xxx

Infusion of buchu...........................℥ iij

Divide into three doses. Take the whole during the day, one-third part at a dose. Keep this up if it acts well, as it will wash out all the old epithelium and tube casts of the kidneys, and render the urine alkaline and the kidneys will act more easily.—*Clinic.*

